# Parliament and the Professions

**Published:** 1921-08-27

**Authors:** 


### Parliament and the Professions.

### Hospital Subscriptions and Income Tax.

Sir R. Horne replied to Captain Bowyer that he cannot see his way to promise legislative proposals in the direction of arranging that next year's subscriptions to hospitals should not be liable to income tax.

### Registration of Nurses.

Replying to a question asked of the Secretary for Scotland by Major Henderson in regard to alleged objections by several of the larger boroughs in Scotland to the Draft Rules of the General Nursing Council for Scotland, Mr. Pratt stated that objection has been taken. The Secretary of State for Scotland considers it most desirable that the registration of existing nurses in Scotland should not be delayed, and as the rules have been carefully adjusted with a view to securing reciprocity of registration with other parts of the kingdom, and as they in no way prejudice the rules that may be made for future nurses, he is not prepared to withdraw them.

